# Effects of green tea polyphenols on inflammation and iron status

**DOI:** 10.1017/jns.2023.107

**Published:** 2023-11-30

**Authors:** Mary Carolyn Jorgenson, Sixtus Aguree, Kevin L. Schalinske, Manju B. Reddy

**Affiliations:** 1 Department of Food Science and Human Nutrition, Iowa State University, Ames, IA, USA; 2 Department of Applied Health Science, Indiana University School of Public Health—Bloomington, Bloomington, IN, USA

**Keywords:** EGCG, Hepcidin, Inflammation, Iron

## Abstract

Inflammation is an underlying problem for many disease states and has been implicated in iron deficiency (ID). This study aimed to determine whether iron status is improved by epigallocatechin-3-gallate (EGCG) through reducing inflammation. Thirty-two male Sprague–Dawley rats were fed an iron-deficient diet for 2 weeks and then randomly divided into four groups (*n* 8 each): positive controls, negative controls, lipopolysaccharide (LPS, 0⋅5 mg/kg body weight), and LPS + EGCG (LPS plus 600 mg EGCG/kg diet) for 3 additional weeks. The study involved testing two control groups, both treated with saline. One group (positive control) was fed a regular diet containing standard iron, while the negative control was fed an iron-deficient diet. Additionally, two treatment groups were tested. The first group was given LPS, while the second group was administered LPS and fed an EGCG diet. Iron status, hepcidin, C-reactive protein (CRP), serum amyloid A (SAA), and interleukin-6 (IL-6) were measured. There were no differences in treatment groups compared with control in CRP, hepcidin, and liver iron concentrations. Serum iron concentrations were significantly lower in the LPS (*P* = 0⋅02) and the LPS + EGCG (*P* = 0⋅01) than in the positive control group. Compared to the positive control group, spleen iron concentrations were significantly lower in the negative control (*P* < 0⋅001) but not with both LPS groups. SAA concentrations were significantly lower in the LPS + EGCG group compared to LPS alone group. EGCG reduced SAA concentrations but did not affect hepcidin or improve serum iron concentration or other iron markers.

## Introduction

Iron deficiency (ID) is a common micronutrient deficiency, affecting approximately 18% of children under 5 years.^(1)^ Iron deficiency anaemia (IDA) occurs when iron metabolism is disrupted and, therefore, cannot fulfil necessary physiological needs or participate in essential biological processes.^(2)^ Hepcidin — a peptide hormone produced in the liver — regulates iron homeostasis^(3,4)^ and is modulated mainly by body iron stores, erythropoiesis, and inflammation.^(5,6)^ Elevated hepcidin levels are linked to reduced intestinal absorption and iron release from the tissues and macrophages, resulting in low circulating serum iron.^(7)^ Patients with true IDA without underlying conditions will exhibit reduced hepcidin concentrations to facilitate iron release into the blood for haemoglobin synthesis. Inflammation and infection can induce hepcidin expression leading to increased body iron storage and reduced plasma iron pool.^(8–13)^ Non-pharmacological approaches that reduce inflammation could improve iron status by downregulating hepcidin expression.

Many dietary components have been shown to reduce inflammation, including foods rich in lycopene and polyphenols.^(14)^ Green tea, abundant in polyphenols, is a popular and widely accessible beverage consumed by much of the general population. Polyphenols are bioactive secondary plant metabolites in fruits and vegetables that contribute to their colour, flavour, and pharmacological activities.^(15)^ Those in green tea are called catechins, including epicatechin, epicatechin-3-gallate, epigallocatechin, and epigallocatechin-3-gallate (EGCG). EGCG is the major catechin in tea, accounting for 50–70 % of catechins in green tea, and it is well-researched for its health benefits.^(16)^ It is best known for its antioxidant and anti-inflammatory properties.^(17)^ As an antioxidant, EGCG has also been shown to increase cell viability by decreasing reactive oxygen species.^(18–20)^ Importantly, Kim *et al.*^(21)^ found that EGCG was effective in preventing IL-8 production, which in turn, reduced the degree of inflammatory response. However, no studies to date have reported improved iron status by reducing inflammation. Given its anti-inflammatory properties, we hypothesised that EGCG would reduce inflammation and thus improve iron status. In the present study, we used lipopolysaccharide (LPS) to induce inflammation in an animal model^(22)^ to study the relationship between obesity-induced inflammation and iron status. The Sprague–Dawley rat is commonly used as an animal model in medical and nutrition research.^(23–25)^ It was chosen for this study because it has been extensively used to study iron metabolism and inflammation.^(26–28)^

Although the relationship between inflammation and iron status has been widely reported, only a few studies have examined these conditions together with a non-pharmacological dietary supplement as an intervention. Understanding the relationship between chronic inflammation and iron status is essential when managing global health issues such as IDA and obesity. Thus, the objectives of this study were to (1) determine whether LPS-induced inflammation will affect iron status and (2) whether EGCG supplementation will suppress LPS-induced inflammation to maintain iron status.

## Materials and methods

### Animal diets and study design

**Fig. 1.**
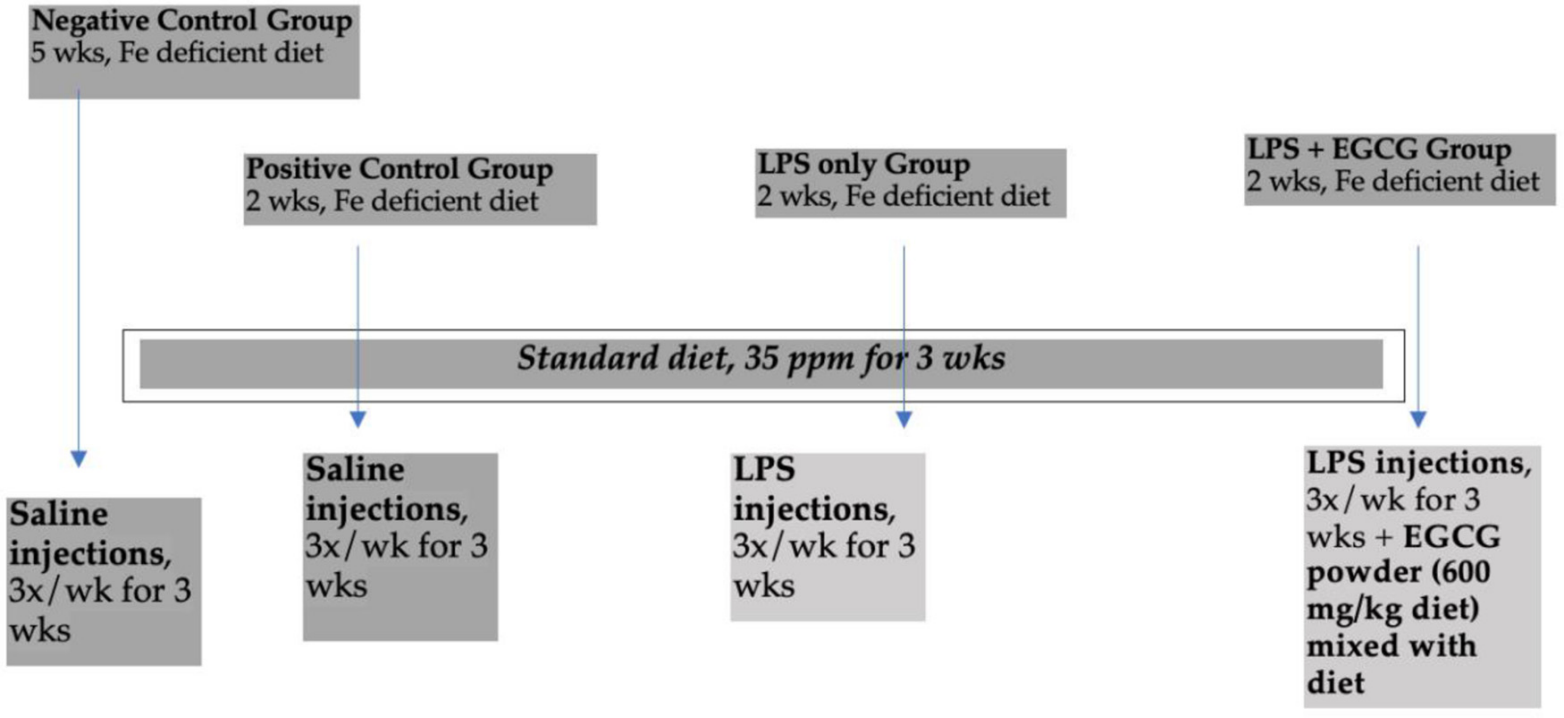
Study design. LPS, lipopolysaccharide; EGCG, epigallocatechin-3-gallate; LPS injections given three times a week for 3 weeks intraperitoneally = 0⋅5 mg/kg body weight.

Our animal study was approved by the Institutional Animal Care and Use Committee at Iowa State University and was performed according to the Iowa State University Laboratory Animal Resources Guidelines. Male Sprague–Dawley rats (*n* 32) were obtained at 21 days of age from Charles River Laboratories (Chicago, IL, USA). After 3 days of acclimation on a standard rat chow, rats were randomly assigned to one of four groups (*n* 8): negative control, positive control, treatment group 1 (LPS only), and treatment group 2 (LPS + EGCG). Previous studies have demonstrated significant changes in iron biomarkers and cellular function in rats who were supplemented with iron for 21 days.^(29–31)^ The use of 6–12 rats is a common practice in iron research to determine the impact of iron on important biomarkers.^(29,32)^ All rats were placed on a powdered iron-deficient diet for 2 weeks (sufficient time to develop iron deficiency^(33)^) at the start of the study: AIN-76A-modified diet containing 2–6 ppm Fe, 20 % casein; 0⋅3 % DL-Methionine; 55 % sucrose; 15 % maize starch; 5 % maize oil, 3⋅5 % mineral mix (iron deficient); 1 % vitamin mix; 0⋅2 % choline bitartrate. After 2 weeks, the positive control and treatment groups were placed on a powdered iron repletion diet, while the negative control remained on the iron-deficient diet. The iron repletion diet contained 35 ppm/kg Fe added as FeSO_4_, 20 % casein; 0⋅3 % dl-Methionine; 55 % sucrose; 15 % maize starch; 5 % maize oil, 3⋅5 % mineral mix; 0⋅02 % ferrous sulphate; 1 % vitamin mix; 0⋅2 % choline bitartrate. Both diets were purchased from Envigo-Teklnad (Indianapolis, IN, USA) and stored at 4 °C until needed. The green tea extract powder contained 50 % of total polyphenols as EGCG and was kindly provided by Givaudan (South Hackensack, NJ, USA). It was mixed thoroughly (600 mg EGCG/kg diet) with the iron-sufficient diet just before use based on dosages reported in other rats studies.^(34)^ The rats were kept in cages that had proper ventilation, and they were housed in a room that was kept at a temperature of 21 ± 1 °C. The room had a consistent pattern of 12 h of light and 12 h of darkness. Food and water were provided ad lib. The detailed study design is shown in Fig. 1.

The negative and positive control groups were given intraperitoneal saline (Sigma Aldrich, St. Louis, MO, USA) injections (0⋅5 ml/kg BW) three times per week. The treatment groups were given intraperitoneal injections of LPS at a dose of 0⋅5 mg/kg BW. We used an intermittent bolus of LPS injection based on a previous study that showed 0⋅5 mg of LPS per kg of body weight produced systemic inflammation with a low risk of fatality.^(22)^ The LPS was derived from *Escherichia coli* O55:B5 and was obtained from Sigma Aldrich (St. Louis, MO, USA). It was dissolved in phosphate-buffered saline and stored at 4 °C until use.

### Institutional review board statement

The animal study protocol was approved by the Institutional Animal Care and Use Committee of Iowa State University (Approval number: IACUC-21–039; approved on 12 April 2021). The Iowa State University's Laboratory Animal Resources (LAR) operation adheres to the U.S. Public Health Service Policy and USDA Animal Welfare Act Regulations in the management and operation of laboratory animals, ensuring humane care and treatment. The animals were given standard rodent chow and access to water at all times.

### Growth assessment and tissue collection

Body weight and food intake were measured daily. After 3 weeks, all the rats were anaesthetised by injecting ketamine (90 mg/kg) /xylazine (10 mg/kg) intraperitoneally). After collecting blood, tissues (liver and spleen) were harvested, rinsed in normal saline solution (0⋅9 %, w/v, NaCl), immediately weighed, and quickly wrapped in labelled foil and snap-frozen and stored at −80 °C until further analysis. The collected blood was allowed to clot, and centrifuged, and the serum was aliquoted and frozen (−80 °C) until subsequent biochemistry was performed. Serum samples were used to measure iron status (haemoglobin, haematocrit, ferritin, and serum iron) and inflammation markers (CRP, IL6, and SAA). The tissue samples were used to measure total iron concentrations.

### Iron status indicators

Whole blood was used immediately to measure haemoglobin (haemocue Hb 201+) concentrations and haematocrit. Serum iron was determined using a commercial kit based on the total iron-binding capacity and serum iron assay kit (Abcam, Waltham, MA, USA). Tissue iron content was measured to assess iron stores using a standard ferrozine assay used in a previous study.^(35)^ Briefly, livers were homogenised in water and subjected to trichloroacetic acid (TCA) protein precipitation at 65 °C for 20 h. Non-haeme iron assay was determined calorimetrically using ferrozine in thioglycolic acid by measuring the absorbance using a microplate reader (BioTek Instruments, Winooski, VT, USA) to assess soluble iron and calculating tissue iron content based on the weight of the tissue used. The same process was used to measure spleen iron content.

### Inflammatory markers

CRP was determined using a commercial kit based on the Rat CRP SimpleStep ELISA kit (Abcam, Waltham, MA, USA). This assay employs an affinity tag-labelled capture antibody and a reporter-conjugated detector antibody, which immunocaptures the sample analyte in solution. This entire complex (capture antibody/analyte/detector antibody) is, in turn, immobilised via immunoaffinity of an anti-tag antibody coating the well. 3,3′5,5′-Tetramethylbenzidine (TMB) solution was added to the sample wells and catalysed by horseradish peroxidase (HRP), and the blue colour intensity was read at 450 nm using a microplate reader. IL-6 concentrations were measured using a commercial kit based on the Rat IL-6 ELISA kit (Millipore, St. Louis, MO, USA) in which the detection antibody was a biotinylated rat IL-6 antibody incubated with HRP + Streptavidin to determine results. SAA (an acute-phase protein and biomarker of inflammation) was measured using a commercial kit using sandwich ELISA (MyBioSource, San Diego, CA, USA). This kit used the pre-coated anti-rat SAA monoclonal antibody, and the detection antibody was a biotinylated polyclonal antibody.

### Statistical analysis

Data were analysed using GraphPad Prism version 9 (La Jolla, CA, USA). Data are expressed as mean ± sem. Statistical differences among the groups were determined using one-way ANOVA with Tukey multiple comparisons test. Differences were considered significant at *P* ≤ 0⋅05.

## Results

The average weight at the beginning of the study was 61 g, then increased to 269 g at the end (Table 1). There were no statistical differences among the groups when weight was compared each week (data not shown). The average daily food intake was 10⋅0 ± 0⋅6 g, with no statistical differences among groups. 600 mg of green tea powder was mixed thoroughly per 1 kg of diet. The average amount of total diet consumed per day for the LPS + EGCG group was approximately 9⋅61 g (Table 1). Thus, we can assume that approximately 5⋅8 mg of green tea powder was consumed per day.

**Table 1.** Final body weights and average daily food intake^a^

Treatment group	Final weight (g)	Daily food intake (g)
Positive control	279 ± 4	10⋅98 ± 0⋅4
Negative control	259 ± 6	9⋅48 ± 0⋅3
LPS	272 ± 5	9⋅94 ± 0⋅7
LPS + EGCG	264 ± 9	9⋅61 ± 0⋅9

a Values are mean ± sem.

**Fig. 2.**
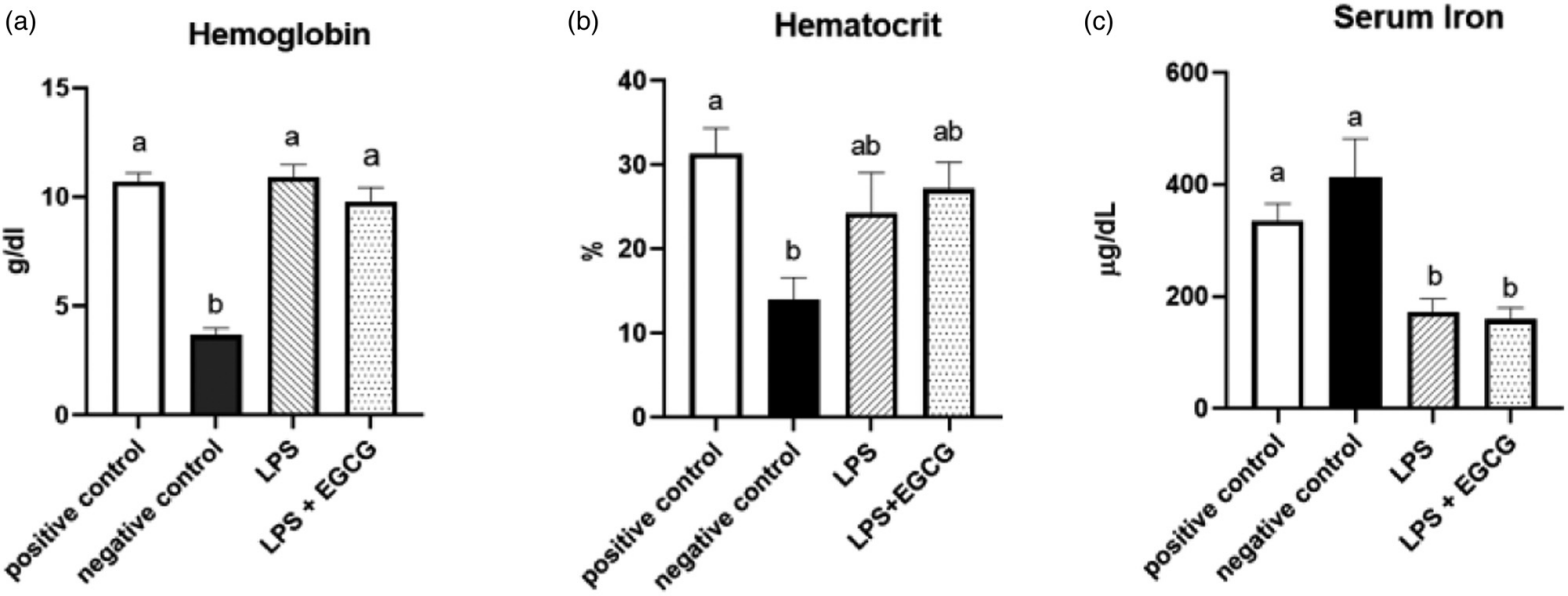
Effect of EGCG on haemoglobin concentrations (a), haematocrit (b), and serum iron (c). Data are presented as mean ± sem, eight per treatment group, and means with different letters are significantly different (*P* < 0⋅05) based on ANOVA with Tukey's multiple comparison test for each measure. *n 7* in the positive control group for haematocrit due to insufficient blood, and *n 7* in both experimental groups due to unexpected rodent deaths early in the study.

As expected, haemoglobin concentration in the negative control group was lower (*P* < 0⋅0001) than in the positive control. In both LPS treatment groups (LPS, 10⋅9 ± 0⋅6 g/dl; LPS + EGCG, 9⋅8 ± 0⋅7 g/dl), haemoglobin concentrations were significantly higher than the negative control group (3⋅7 ± 0⋅3 g/dl) but no statistical differences compared to positive control (10⋅7+ ± 0⋅4). We observed the same trend with haematocrit values. The negative control (13⋅9 ± 2⋅9 %) was significantly (*P* < 0⋅005) lower than the positive control (31⋅4 ± 3⋅3 %). The values in LPS (24⋅3 ± 5⋅2 %) and LPS + EGCG (27⋅2 ± 3⋅4 %) were not significantly different from each other or with the positive or negative control groups. Overall, EGCG treatment had no effect on haemoglobin and haematocrit—as noted by comparable estimates between LPS and LPS + EGCG groups (Fig. 2(a), (b)).

Interestingly, feeding an iron-deficient diet did not significantly reduce serum iron concentrations in the negative control. However, inducing inflammation with LPS significantly reduced serum iron compared to positive control. Compared to the positive control (335⋅8 ± 32⋅1 μg/dL), serum iron concentrations (mean ± sem) were 50 % lower in the LPS only (172⋅5 ± 25⋅1 μg/dL) (*P* = 0⋅02) and the LPS + EGCG (159⋅80 ± 22⋅34 μg/dL) (*P* = 0⋅01) groups. There was no significant difference in serum iron concentration between the LPS only group and the LPS + EGCG group, indicating that EGCG consumptionhad no effect on serum iron concentrations (Fig. 2(c)). Two rodents, one from each treatment group, died after receiving one and two LPS injections, respectively. We believe these fatalities could be attributed to the LPS dosage utilised in the research.

**Fig. 3.**
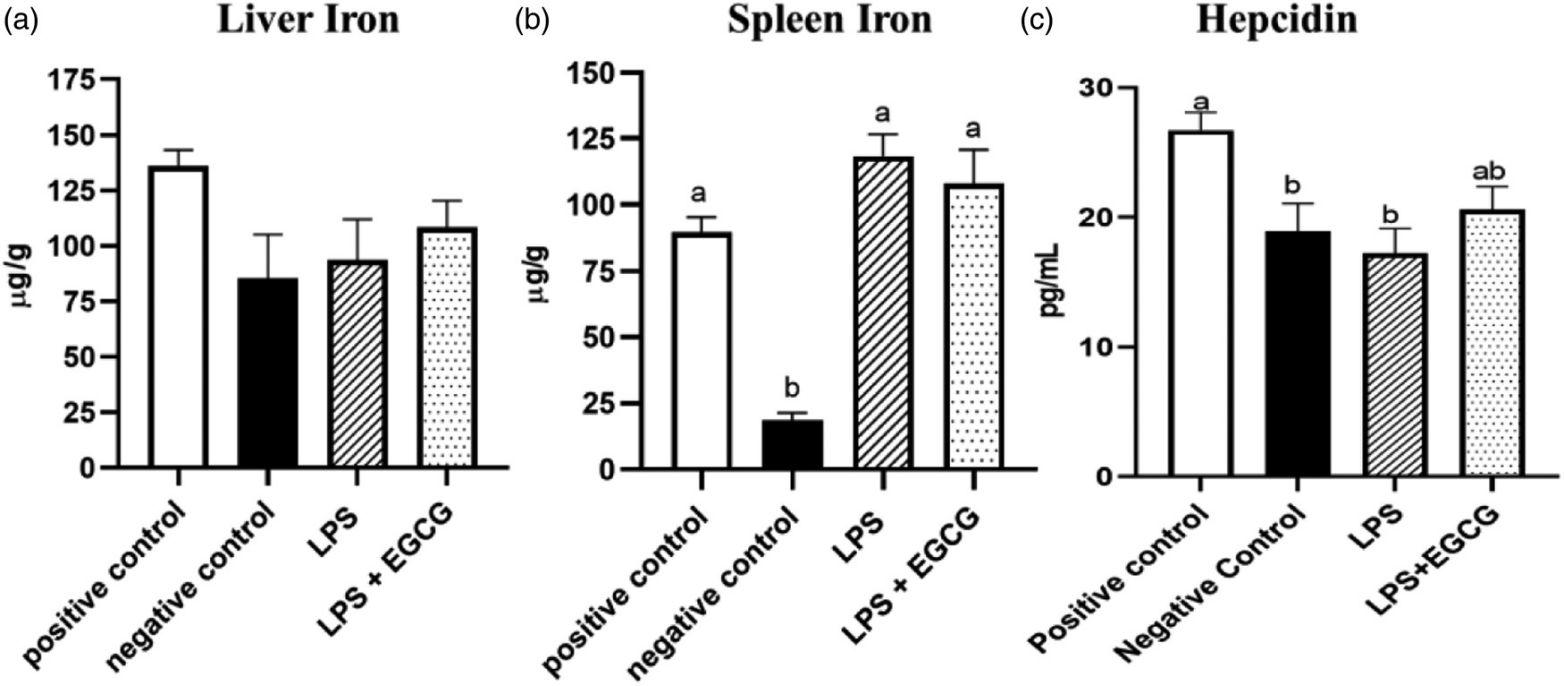
Effects of EGCG and inflammation on liver (a) and spleen iron concentrations (b) and hepcidin concentrations (c). Data are presented as mean ± sem, eight per treatment group(*n* 7 in both experimental groups due to unexpected rodent deaths early in the study). Mean scores with different letters indicate statistical difference (*P* < 0⋅05), based on Tukey's multiple comparison test.

Though there were no significant differences in liver iron concentrations (Fig. 3(a)) among the groups, spleen iron concentrations were significantly lower in the negative control (18⋅6 ± 2⋅8 μg/g) (*P* < 0⋅001) compared to the positive control group (89⋅6 ± 5⋅7 μg/g). Both LPS treatment groups had significantly higher iron content than the negative control, but no significant differences were observed between LPS (118⋅4 ± 8⋅3 μg/g) and LPS + EGCG (108⋅1 ± 12⋅8 μg/g) (Fig. 3(b)). As expected, hepcidin concentrations in the negative control (*P* < 0⋅05) and LPS-only groups (*P* = 0⋅01) were significantly lower than in the positive control. It appears that feeding rats with EGCG had no effect on hepcidin concentrations, as there was no difference between the two treatment groups (Fig. 3(c)).

**Fig. 4.**
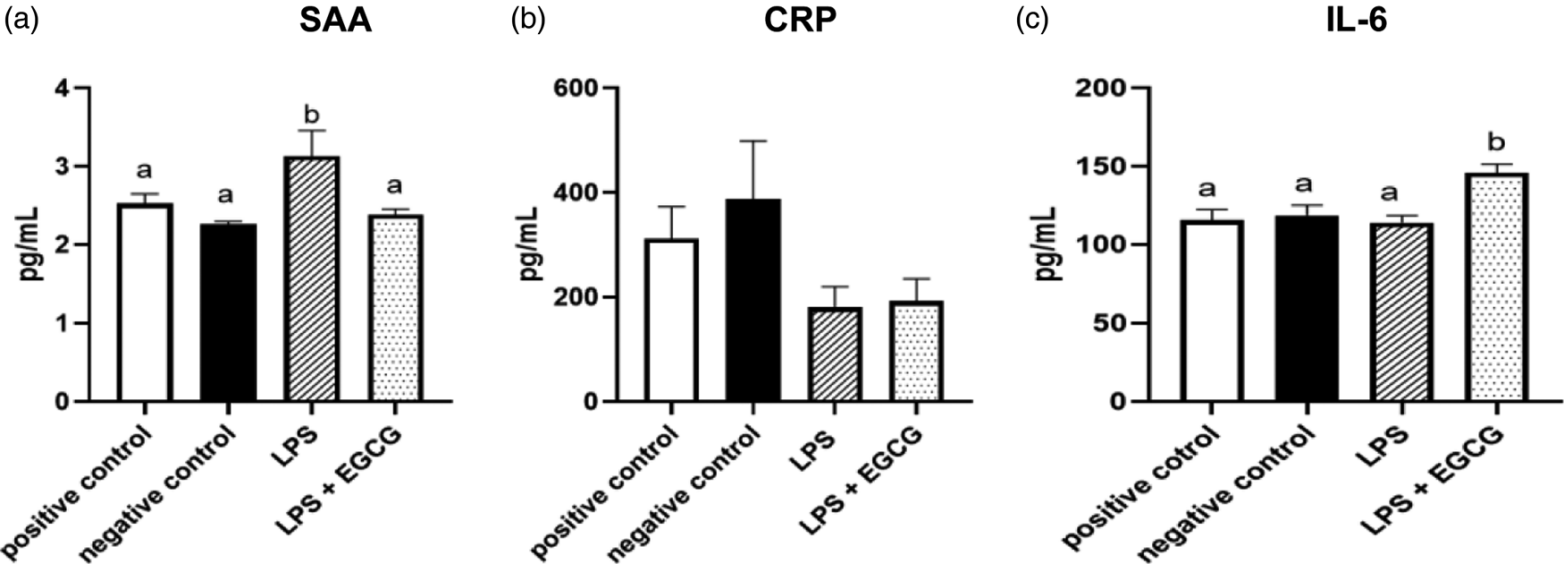
Effects of EGCG on inflammation markers. Serum amyloid A (SAA) (a), C-reactive protein (CRP) (b), IL-6 concentrations (c), data are presented as mean ± sem, eight per treatment group (*n 7* in both experimental groups due to unexpected rodent deaths early in the study). Mean scores with different letters are significantly different (*P* < 0⋅05) based on ANOVA with Tukey's multiple comparison test for each measure.

When observing inflammatory markers, SAA concentrations were significantly higher in LPS only group (3⋅1 ± 0⋅4 ng/ml) (*P* = 0⋅01) compared to the negative control group(2⋅3 ± 0⋅03 ng/ml) (Fig. 4(a)). Feeding EGCG significantly (*P* < 0⋅05) lowered SAA concentrations to a level similar to the negative and positive control groups suggesting reduced inflammation. Surprisingly, no significant differences were observed in CRP concentrations among the groups (Fig. 4(b)). IL-6 concentrations were significantly higher in the LPS + EGCG group than in all the groups. Unexpectedly, the LPS + EGCG group had significantly (146⋅1 ± 6⋅0 pg/ml *P* = 0⋅01) higher IL-6 concentrations than LPS alone (114⋅33 ± 4⋅78 pg/ml) group. (Fig. 4(c)).

## Discussion

Previous studies have shown a relationship between inflammatory markers and IDA,^(8,36)^ but no studies till date propose using a dietary component to counteract inflammatory response in order to improve iron status. In the present study, LPS injection resulted in reduced serum iron but feeding rats with EGCG had no effect in counteracting inflammatory responses induced by LPS. However, the elevated SAA concentrations with LPS were significantly reduced by EGCG, suggesting its anti-inflammatory property.

The results of our study were unexpected as we did not observe any significant differences in cytokines or inflammation between the control group and the group treated with LPS. This contrasts with previous research which found that LPS administration increased levels of cytokines, particularly IL-6, and other markers of inflammation.^(22)^

However, it is worth noting that the previous study used higher doses of LPS (1⋅0 ml/kg BW and 2 ml/kg BW), resulting in higher mortality rates than the lower dose used in our study (0⋅5 ml/kg BW). Our study had a higher survival rate, but stronger inflammatory responses and changes in hepcidin expression may require higher doses of LPS. Another difference between the two studies is the age of the rats used. The previous study used rats that were 9 weeks old when the LPS treatment began and over 13 weeks old at the end of the experiment, whereas our rats were only 5 weeks old at the start and 8 weeks old at the end. This is because it has been reported in mice that the levels of pro-inflammatory of TNF-α and IL-1β in were higher in mature compared to young control group following LPS administration.^(37)^ This difference in maturity could explain the varying inflammatory responses and the lack of difference between the treatment and control groups in our study. As the inflammatory responses were comparable between treatment groups, it was unsurprising that there were no significant differences in either experimental group when comparing haemoglobin concentrations and haematocrit to the positive control. The low haemoglobin concentrations in the negative control indicate that the rats were indeed iron deficient, thus providing knowledge for future studies to allow more focus on reducing chronic inflammation rather than inducing iron deficiency.

We also anticipated significant differences among liver iron concentrations, but no such findings were observed. Based on the reduction of serum iron following LPS-induced inflammation (based on SAA concentrations), we also expected high iron stores in the liver, but this was not observed. However, the LPS-only group showed a significant increase in spleen iron concentrations compared to the negative control. This could indicate ‘iron trapping,’ which is driven by hepcidin. Increased hepcidin levels will lead to an increase in endocytosis and the breakdown of ferroportin, thus keeping iron trapped within cells. This reduces the natural flow of iron from hepatocytes, enterocytes, and macrophages resulting in reduced serum iron concentration.^(38)^ Compared to the positive control, serum iron concentrations were 50 % lower in the LPS only and the LPS + EGCG groups. The levels of IL-6 and hepcidin were consistent among all groups,, but it is still uncertain what caused the decrease in serum concentration in the LPS group. Measuring other cytokines and inflammatory biomarkers might help to provide some clues to explain the observed changes in serum iron concentration. This indicates that EGCG did not improve serum iron, although some reduction in inflammation was found in terms of SAA concentration. However, the lower serum iron in the LPS only and LPS + EGCG groups demonstrates iron trapping theory in inflammation. The low serum iron was likely in response to inflammation, as it is consistent with previous studies that focused on inflammatory biomarkers.^(39,40)^

Higher concentrations of SAA in the LPS-only group but not in the LPS + EGCG group support our hypothesis. This data point alone indicates that the EGCG did reduce inflammation. The IL-6 data contradict this, as concentrations were elevated in the LPS + EGCG group but not in the LPS-only group or negative control. However, IL-6 concentrations are elevated in LPS + EGCG, which might be an anti-inflammatory response to ECCG as IL-6 also has extensive anti-inflammatory functions as a myokine.^(40)^ Overall, inflammatory markers as a whole were inconclusive.

The inconclusive inflammatory biomarkers could also be due, in part, to the short half-life of CRP, which is approximately 19 h.^(41)^ As an acute-phase protein, it has been reported that the plasma concentration of CRP deviates by at least 25 % during inflammatory conditions.^(42)^ Importantly, when there is no longer a stimulus, CRP values reportedly decrease over 18–20 h.^(43)^ Thus, the timing of the blood draws in relation to the final dose of LPS may have been too large a time gap as other studies have reported a peak of pro-inflammatory cytokines occurring between 2 and 6 h post LPS administration.^(44,45)^ As the blood samples and tissues were collected a day after the administration of LPS, any changes in pro-inflammatory cytokines would have resolved before sample collection. We believe this to be the case with hepcidin, as it is also a transient hormone with a reported half-life of 2⋅3 min^(46)^ Thus, a blood draw closer to the time of LPS injection may have produced the expected hepcidin results. Importantly, previous studies suggest inflammatory regulators take precedence over iron stores: iron-deficient mice injected with LPS up-regulated hepcidin expression,^(47)^ while iron-loaded mice with experimentally induced anaemia down-regulated hepcidin expression.^(48)^ These contradictory assessment factors caused by inflammation could also explain the insignificant results observed in the liver iron.

The rationale behind using LPS was to induce inflammation similar to that of obesity, and it has been reported that inflammation is linked to increased adiposity.^(49)^ IDA cannot be differentiated from the more prominent anaemia of inflammation because inflammation confounds the measurement of iron status.^(8)^ Stoffel *et al.*^(50)^ evaluated different iron and inflammatory biomarkers in normal-weight *v.* obese women. They found higher levels of central adiposity correlated with elevated CRP, α-1 glycoprotein, serum hepcidin, total iron-binding capacity, and lower serum iron-to-hepcidin ratio and transferrin saturation.^(50)^ Similar results were reported by our group when normal-weight and obese subjects were compared to obese subjects.^(8)^ The CRP values in the obese group were 8 times higher than in normal-weight women, and most of the normal-weight subjects were within the normal range, while those of the obese group were elevated.^(8)^

There were many strengths and limitations to this research. Measuring inflammatory markers such as CRP and hepcidin at a more optimal time could provide a clearer picture of whether inflammation was present due to LPS treatment. Measurement of iron biomarkers at the end of the iron-depletion phase just before the randomisation could also provide definitive answers as to whether the rats were indeed iron deficient prior to LPS and or EGCG treatment. Despite this limitation, the findings of this study are important because we measured a range of physiological factors that affect iron status and have opened the door to determining more ways to treat anaemia and chronic inflammation. Future studies could include a different LPS and EGCG administration regimen and dosage. Such studies should also measure more cytokines (e.g. tumour necrosis factor-α (TNF-α), interferon (IFN)-γ, interleukin-1 (IL-1), IL-1α, IL-1β), inflammatory, and iron biomarkers, as this will provide an opportunity to assess the effect of LPS and EGCG administration on iron metabolism. However, it should be noted that in the present study, we took a cautious approachfor each regimen to minimize stress on the rodents. It is possible that if we had used a higher dose of LPS administration or studied chronic inflammation (such as obesity), the results may have been different.

## Conclusions

Multiple doses of 0⋅5 ml/kg of LPS did not increase serum cytokines (IL-6), CRP, or hepcidin. Higher doses of LPS may be needed to induce increased expression of pro-inflammatory cytokine changes in young rats. Blood and tissue samples should be collected a few hours after LPS administration to observe changes in cytokine and hepcidin concentrations.

## References

[1] Gedfie, Getawa S & Melku M. Prevalence and associated factors of iron deficiency and iron deficiency anemia among under-5 children: a systematic review and meta-analysis. Glob Pediatr Health 2022;6(9). doi: 10.1177/2333794X221110860. PMC9272181 35832654

[2] Zimmermann MB & Hurrell RF. Nutritional iron deficiency. Lancet. 2007;370(9586):511–520. doi: 10.1016/S0140-6736(07)61235-5. 17693180

[3] Nemeth E & Ganz T. The role of hepcidin in iron metabolism. Acta Haematol. 2009;122(2–3):78–86. doi: 10.1159/000243791. 19907144 PMC2855274

[4] Ganz T. Hepcidin, a key regulator of iron metabolism and mediator of anemia of inflammation. Blood. 2003;102(3):783–788. doi: 10.1182/blood-2003-03-0672. 12663437

[5] Hentze MW, Muckenthaler MU, Galy B, et al. Two to tango: regulation of mammalian iron metabolism. Cell. 2010;142(1):24–38. doi: 10.1016/j.cell.2010.06.028. 20603012

[6] Camaschella C & Pagani A. Advances in understanding iron metabolism and its crosstalk with erythropoiesis. Br J Haematol. 2018;182(4):481–494. doi: 10.1111/bjh.15403. 29938779

[7] Nemeth E, Tuttle MS, Powelson J, et al. Hepcidin regulates cellular iron efflux by binding to ferroportin and inducing its internalization. Science. 2004;306(5704):2090–2093. doi: 10.1126/science.1104742. 15514116

[8] Aguree S & Reddy MB. Inflammatory markers and hepcidin are elevated but serum iron is lower in obese women of reproductive age. Nutrients. 2021;13(1). doi: 10.3390/nu13010217. PMC7828682 33466578

[9] Zimmermann MB, Zeder C, Muthayya S, et al. Adiposity in women and children from transition countries predicts decreased iron absorption, iron deficiency and a reduced response to iron fortification. Int J Obes (Lond). 2008;32(7):1098–110. doi: 10.1038/ijo.2008.43. 18427564

[10] Aeberli I, Hurrell RF & Zimmermann MB. Overweight children have higher circulating hepcidin concentrations and lower iron status but have dietary iron intakes and bioavailability comparable with normal weight children. Int J Obes (Lond). 2009;33(10):1111–1117. doi: 10.1038/ijo.2009.146. 19636315

[11] Mujica-Coopman MF, Brito A, López de Romaña D, et al. Body mass index, iron absorption and iron status in childbearing age women. J Trace Elem Med Biol. 2015;30:215–219. doi: 10.1016/j.jtemb.2014.03.008. 24813452

[12] Laftah AH, Ramesh B, Simpson RJ, et al. Effect of hepcidin on intestinal iron absorption in mice. Blood. 2004;103(10):3940–3944. doi: 10.1182/blood-2003-03-0953. 14751922

[13] Cepeda-Lopez AC, Aeberli I & Zimmermann MB. Does obesity increase risk for iron deficiency? A review of the literature and the potential mechanisms. Int J Vitam Nutr Res. 2010;80(4–5):263–270. doi: 10.1024/0300-9831/a000033. 21462109

[14] Ghavipour M, Saedisomeolia A, Djalali M, et al. Tomato juice consumption reduces systemic inflammation in overweight and obese females. Br J Nutr. 2013;109(11):2031–2035. doi: 10.1017/S0007114512004278. 23069270

[15] Recio MC, Andujar I & Rios JL. Anti-inflammatory agents from plants: progress and potential. Curr Med Chem. 2012;19(14):2088–2103. doi: 10.2174/092986712800229069. 22414101

[16] Khan N & Mukhtar H. Tea polyphenols in promotion of human health. Nutrients. 2018;11(1). doi: 10.3390/nu11010039. PMC6356332 30585192

[17] Chu C, Deng J, Man Y, et al. Green tea extracts epigallocatechin-3-gallate for different treatments. Biomed Res Int. 2017;2017:5615647. doi: 10.1155/2017/5615647. 28884125 PMC5572593

[18] Zhang X, Wu M, Lu F, et al. Involvement of α7 nAChR signaling cascade in epigallocatechin gallate suppression of β-amyloid-induced apoptotic cortical neuronal insults. Mol Neurobiol. 2014;49(1):66–77. doi: 10.1007/s12035-013-8491-x. 23807728

[19] Higdon JV & Frei B. Tea catechins and polyphenols: health effects, metabolism, and antioxidant functions. Crit Rev Food Sci Nutr. 2003;43(1):89–143. doi: 10.1080/10408690390826464. 12587987

[20] Frei B & Higdon JV. Antioxidant activity of tea polyphenols in vivo: evidence from animal studies. J Nutr. 2003;133(10):3275s–32784s. doi: 10.1093/jn/133.10.3275S. 14519826

[21] Kim IB, Kim DY, Lee SJ, et al. Inhibition of IL-8 production by green tea polyphenols in human nasal fibroblasts and A549 epithelial cells. Biol Pharm Bull. 2006;29(6):1120–1125. doi: 10.1248/bpb.29.1120. 16755003

[22] Ranneh Y, Akim AM, Hamid HA, et al. Induction of chronic subclinical systemic inflammation in sprague-dawley rats stimulated by intermittent bolus injection of lipopolysaccharide. Arch Immunol Ther Exp (Warsz). 2019;67(6):385–400. doi: 10.1007/s00005-019-00553-6. 31278602

[23] Piñero DJ, Li N, Hu J, et al. The intracellular location of iron regulatory proteins is altered as a function of iron status in cell cultures and Rat brain. J Nutr. 2001;131(11):2831–2836. doi: 10.1093/jn/131.11.2831. 11694604

[24] Turbino-Ribeiro SML, Silva ME, Chianca DA Jr., et al. Iron overload in hypercholesterolemic rats affects iron homeostasis and serum lipids but not blood pressure. J Nutr. 2003;133(1):15–20. doi: 10.1093/jn/133.1.15. 12514260

[25] Benson VL, McMahon AC, Lowe HC, et al. The streptozotocin-treated sprague-dawley rat: a useful model for the assessment of acute and chronic effects of myocardial ischaemia reperfusion injury in experimental diabetes. Diab Vasc Dis Res. 2007;4(2):153–154. doi: 10.3132/dvdr.2007.035. 17654451

[26] Uritski R, Bilkis I, Reifen R, et al. Dietary iron affects inflammatory status in a rat model of colitis. J Nutr. 2004;134(9):2251–2255. doi: 10.1093/jn/134.9.2251. 15333712

[27] Lyoumi S, Tamion F, Petit J, et al. Induction and modulation of acute-phase response by protein malnutrition in rats: comparative effect of systemic and localized inflammation on interleukin-6 and acute-phase protein Synthesis^1,2^. J Nutr. 1998;128(2):166–174. doi: 10.1093/jn/128.2.166. 9446838

[28] Sword JT, Pope AL & Hoekstra WG. Endotoxin and lipid peroxidation in vitro in selenium- and vitamin E-deficient and -adequate rat tissues. J Nutr. 1991;121(2):258–264. doi: 10.1093/jn/121.2.258. 1995794

[29] Ross KL & Eisenstein RS. Iron deficiency decreases mitochondrial aconitase abundance and citrate concentration without affecting tricarboxylic acid cycle capacity in rat liver. J Nutr. 2002;132(4):643–651. doi: 10.1093/jn/132.4.643. 11925455

[30] Knutson MD, Walter PB, Ames BN, et al. Both iron deficiency and daily iron supplements increase lipid peroxidation in rats. J Nutr. 2000;130(3):621–628. doi: 10.1093/jn/130.3.621. 10702595

[31] Walter PB, Knutson MD, Paler-Martinez A, et al. Iron deficiency and iron excess damage mitochondria and mitochondrial DNA in rats. Proc Natl Acad Sci U S A. 2002;99(4):2264–2269. doi: 10.1073/pnas.261708798. 11854522 PMC122353

[32] Unger EL, Wiesinger JA, Hao L, et al. Dopamine D2 receptor expression is altered by changes in cellular iron levels in PC12 cells and rat brain tissue. J Nutr. 2008;138(12):2487–2494. doi: 10.3945/jn.108.095224. 19022977 PMC3415866

[33] Zhang AS, Anderson SA, Meyers KR, et al. Evidence that inhibition of hemojuvelin shedding in response to iron is mediated through neogenin. J Biol Chem. 2007;282(17):12547–12556. doi: 10.1074/jbc.M608788200. 17331953

[34] Devika PT & Stanely Mainzen Prince P. Protective effect of (-)-epigallocatechin-gallate (EGCG) on lipid peroxide metabolism in isoproterenol induced myocardial infarction in male wistar rats: a histopathological study. Biomed Pharmacother. 2008;62(10):701–708. doi: 10.1016/j.biopha.2007.10.011. 18078734

[35] Swain JH, Tabatabai LB & Reddy MB. Histidine content of low-molecular-weight beef proteins influences nonheme iron bioavailability in caco-2 cells. J Nutr. 2002;132(2):245–251. doi: 10.1093/jn/132.2.245. 11823585

[36] McClung JP & Karl JP. Iron deficiency and obesity: the contribution of inflammation and diminished iron absorption. Nutr Rev. 2009;67(2):100–104. doi: 10.1111/j.1753-4887.2008.00145.x. 19178651

[37] Zhao YF, Qiong Z, Zhang JF, et al. The synergy of aging and LPS exposure in a mouse model of Parkinson's disease. Aging Dis. 2018;9(5):785–797. doi: 10.14336/AD.2017.1028. 30271656 PMC6147589

[38] Ueda N & Takasawa K. Impact of inflammation on ferritin, hepcidin and the management of iron deficiency anemia in chronic kidney disease. Nutrients. 2018;10(9). doi: 10.3390/nu10091173. PMC6163440 30150549

[39] Suchdev PS, Williams AM, Mei Z, et al. Assessment of iron status in settings of inflammation: challenges and potential approaches. Am J Clin Nutr. 2017;106(Suppl 6):1626s–1633s. doi: 10.3945/ajcn.117.155937. 29070567 PMC5701714

[40] Nara H & Watanabe R. Anti-inflammatory effect of muscle-derived interleukin-6 and its involvement in lipid metabolism. Int J Mol Sci. 2021;22(18). doi: 10.3390/ijms22189889. PMC8471880 34576053

[41] Pepys MB & Hirschfield GM. C-reactive protein: a critical update. J Clin Invest. 2003;111(12):1805–1812. doi: 10.1172/JCI18921. 12813013 PMC161431

[42] Gabay C & Kushner I. Acute-phase proteins and other systemic responses to inflammation. N Engl J Med. 1999;340(6):448–454. doi: 10.1056/NEJM199902113400607. 9971870

[43] Ridker PM. Clinical application of C-reactive protein for cardiovascular disease detection and prevention. Circulation. 2003;107(3):363–369. doi: 10.1161/01.cir.0000053730.47739.3c. 12551853

[44] Matsuzaki J, Kuwamura M, Yamaji R, et al. Inflammatory responses to lipopolysaccharide Are suppressed in 40 % energy-restricted mice. J Nutr. 2001;131(8):2139–2144. doi: 10.1093/jn/131.8.2139. 11481408

[45] Somann JP, Wasilczuk KM, Neihouser KV, et al. Characterization of plasma cytokine response to intraperitoneally administered LPS & subdiaphragmatic branch vagus nerve stimulation in rat model. PLoS One. 2019;14(3):e0214317. doi: 10.1371/journal.pone.0214317. 30921373 PMC6438475

[46] Xiao JJ, Krzyzanski W, Wang YM, et al. Pharmacokinetics of anti-hepcidin monoclonal antibody Ab 12B9m and hepcidin in cynomolgus monkeys. Aaps J. 2010;12(4):646–657. doi: 10.1208/s12248-010-9222-0. 20737261 PMC2977007

[47] Constante M, Jiang W, Wang D, et al. Distinct requirements for Hfe in basal and induced hepcidin levels in iron overload and inflammation. Am J Physiol Gastrointest Liver Physiol. 2006;291(2):G229–G237. doi: 10.1152/ajpgi.00092.2006. 16565419 PMC2891007

[48] Nicolas G, Chauvet C, Viatte L, et al. The gene encoding the iron regulatory peptide hepcidin is regulated by anemia, hypoxia, and inflammation. J Clin Invest. 2002;110(7):1037–1044. doi: 10.1172/JCI15686. 12370282 PMC151151

[49] Welsh P, Polisecki E, Robertson M, et al. Unraveling the directional link between adiposity and inflammation: a bidirectional Mendelian randomization approach. J Clin Endocrinol Metab. 2010;95(1):93–99. doi: 10.1210/jc.2009-1064. 19906786 PMC2805500

[50] Stoffel NU, El-Mallah C, Herter-Aeberli I, et al. The effect of central obesity on inflammation, hepcidin, and iron metabolism in young women. Int J Obes (Lond). 2020;44(6):1291–1300. doi: 10.1038/s41366-020-0522-x. 31974407

